# The Influence of Serious Extracranial Injury on In-Hospital Mortality in Children with Severe Traumatic Brain Injury

**DOI:** 10.3390/jpm12071075

**Published:** 2022-06-30

**Authors:** Min Chao, Chia-Cheng Wang, Carl P. C. Chen, Chia-Ying Chung, Chun-Hsiang Ouyang, Chih-Chi Chen

**Affiliations:** 1 Department of Physical Medicine and Rehabilitation, Chang Gung Memorial Hospital, Chang Gung University, Taoyuan 33305, Taiwan; emily821102@gmail.com (M.C.); carlchendr@gmail.com (C.P.C.C.); chiaying928@yahoo.com (C.-Y.C.); 2 Department of Traumatology and Emergency Surgery, Chang Gung Memorial Hospital, Chang Gung University, Taoyuan 33305, Taiwan; m0827@cgmh.org.tw (C.-C.W.); detv090@gmail.com (C.-H.O.)

**Keywords:** severe traumatic brain injury, children, serious extracranial injury, mortality, predictors

## Abstract

(1) Background: Severe traumatic brain injury (sTBI) is the leading cause of death in children. Serious extracranial injury (SEI) commonly coexists with sTBI after the high impact of trauma. Limited studies evaluate the influence of SEI on the prognosis of pediatric sTBI. We aimed to analyze SEI’s clinical characteristics and initial presentations and evaluate if SEI is predictive of higher in-hospital mortality in these sTBI children. (2) Methods: In this 11-year-observational cohort study, a total of 148 severe sTBI children were enrolled. We collected patients’ initial data in the emergency department, including gender, age, mechanism of injury, coexisting SEI, motor components of the Glasgow Coma Scale (mGCS) score, body temperature, blood pressure, blood glucose level, initial prothrombin time, and intracranial Rotterdam computed tomography (CT) score of the first brain CT scan, as potential mortality predictors. (3) Results: Compared to sTBI children without SEI, children with SEI were older and more presented with initial hypotension and hypothermia; the initial lab showed more prolonged prothrombin time and a higher in-hospital mortality rate. Multivariate analysis showed that motor components of mGCS, fixed pupil reaction, prolonged prothrombin time, and higher Rotterdam CT score were independent predictors of in-hospital mortality in sTBI children. SEI was not an independent predictor of mortality. (4) Conclusions: sTBI children with SEI had significantly higher in-hospital mortality than those without. SEI was not an independent predictor of mortality in our study. Brain injury intensity and its presentations, including lower mGCS, fixed pupil reaction, higher Rotterdam CT score, and severe injury-induced systemic response, presented as initial prolonged prothrombin time, were independent predictors of in-hospital mortality in these sTBI children.

## 1. Introduction

Traumatic brain injury (TBI) is the leading cause of death in children, affecting pediatric patients worldwide [1,2,3,4,5,6,7,8,9,10,11]. Early recognition of children with a high risk of mortality is valuable for clinicians and family members to guide the therapeutic options, especially in children with severe TBI (sTBI).

Coexisting serious extracranial injury (SEI) commonly occurs in TBI patients who suffer high impact injury and has been reported with a prevalence of 23% to 41%, according to different studied populations and definitions of SEI. In sTBI children, the incidence of SEI ranged from 46% to 63% [12,13,14]. Although many studies had suggested SEI have a negative effect on TBI patients’ outcome, most of these studies focused on a wide range of ages of TBI patients and were still with inconclusive consensus [15]. Limited studies evaluate SEI on the prognosis of pediatric TBI groups, and their results were also different. Two of the studies suggested no statistical differences in in-hospital mortality between sTBI with and without SEI but increased morbidity in those with SEI [12,14]. Another study suggested that SEI was independently associated with higher mortality [13].

The association between coexisting SEI and mortality in sTBI children is still inconclusive. The aim of the present study is to analyze the epidemiological and clinical characteristics of SEI-related sTBI children. We also want to evaluate further if SEI is associated with higher mortality in pediatric sTBI children. The goal of this study was to demonstrate the different presentations of sTBI with and without SEI and improve prognostic counseling and further care for these children.

## 2. Materials and Methods

### 2.1. Study Design

We conducted a retrospective, observational cohort study in a tertiary trauma center in Taiwan. Children aged 0–18 years old who were admitted between April 2008 and December 2019 with TBI. Patients were eligible if they had been assigned the International Classification of Diseases, Ninth Edition (ICD-9), diagnostic codes 850–854 for intracranial injury. At admission, GCS score and Abbreviated injury scale (AIS) were routinely scored by emergency medical service personnel. Brain injury severity was stratified by the initial GCS score, which ranges from 3 to 15. sTBI was defined by an initial GCS score ≤ 8. We only recruited children with sTBI in this study. Patients who died at the scene or during transportation were excluded. Those who presented to the emergency department greater than 24 h post injury were also excluded since we wanted to analyze the initial predictors of in-hospital mortality in these patients. Penetrating head injuries were also excluded from this study.

SEI was defined by an AIS score ≥ 3 for the face, chest, abdomen, skin, and extremities [16]. We divided the recruited sTBI children into two groups according to their coexisting SEI or not. A physician and a research nurse reviewed all medical records. Medical histories were gathered and reviewed from patient charts. The Institutional Review Board at Chang Gung Memorial Hospital approved this study: IRB no. 20200050B0.

### 2.2. Variable Definitions

Data were extracted from the medical records of all eligible subjects. Patients were divided into two groups according to the presence of SEI or not. In-hospital mortality was defined as the outcome. The parameters selected to evaluate differences between sTBI patients with or without SEI included age, gender, mechanism of injury, initial clinical presentation to the ED including motor component of GCS score (mGCS score), hypotension, hypothermia, pupil size, pupil reaction, initial laboratory data including hyperglycemia and a prolonged prothrombin time, and intracranial CT findings. Parameters selected to evaluate differences between sTBI children with and without mortality were the same as above and added the parameter of co-existed SEI or not. We selected these reliable parameters based on previous studies that suggested potential risk factors for injury severity and mortality in pediatric TBI [17].

We used the mGCS score instead of the full GCS score as the risk factor of outcome as a previous study has shown that the mGCS score is equivalent to the full GCS score for predicting survival to hospital discharge in sTBI children whose eye and verbal components are difficult to reliably obtain [18]. The mGCS was scored from 1 to 6. Pupil size was defined as 2 points if both pupils were larger than 4 mm; 1 point if one pupil was larger than 4 mm and the other not; 0 if both pupils were smaller than 4 mm [19,20]. Pupil reaction was defined as 2 points when both pupils could not constrict with light shone into either eye alone; 1 point when one of both pupils constricted in reaction to light; and 0 point if both pupils constricted in response to light [20]. Hypotension was diagnosed when a patient’s systolic blood pressure (SBP) was below the fifth percentile for their age. Blood pressure less than 70 mmHg + (2* age in years) in children aged 1 to 10 years old, and less than 90 mmHg in children ≥10 years of age, is defined as hypotension, according to the American Heart Association for Cardiopulmonary Resuscitation and Emergency Cardiovascular Care [21]. Children with an initial body temperature below 35 °C were defined as presenting with hypothermia. [22] Hyperglycemia was defined as a blood glucose level greater than 200 mg/dL on admission to the ED [23], and a prolonged prothrombin time was defined as an international normalized ratio (INR) ≥ 1.2 [24]. A physician blinded to the outcome reviewed the CT images obtained in the first twenty-four hours for each patient and assigned a Rotterdam CT score [25].

### 2.3. Statistical Analysis

We compared the differences between sTBI children with and without SEI in clinical characteristics and outcome variables using descriptive statistics. We used the Mann–Whitney U test to compare continuous variables. Comparisons were made by Pearson’s chi-square test if any expected cell size was less than 5 or Fisher’s exact test for categorical variables. We assumed missing data were completely at random, therefore, multiple imputations were used to estimate the missing data. 

To identify potential predictors of in-hospital mortality among children with severe TBI, we conducted multivariate logistic regression analyses. We used univariate analysis to identify the candidate predictors for in-hospital mortality in sTBI children with a significance level of *p* < 0.1, and the final multivariate model included only statistically significant predictors with *p* < 0.05. Data were entered and analyzed using the STATA version 14.0 software (STATA, Inc., College Station, TX, USA).

## 3. Results

**Figure 1:**
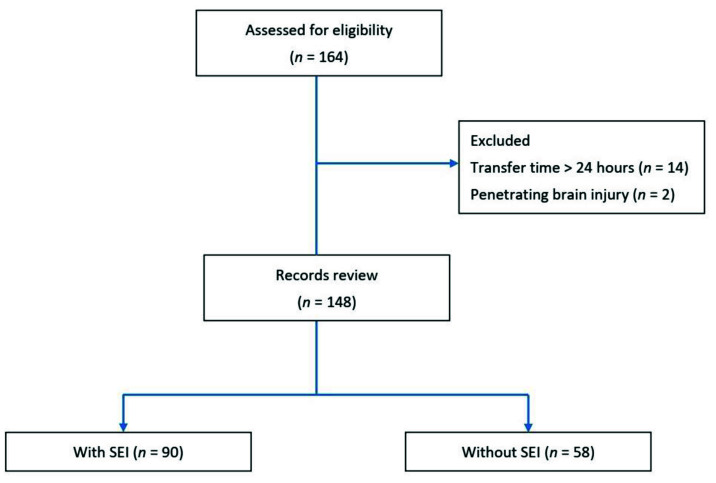
Inclusion and Exclusion flow chart.

**Figure 2:**
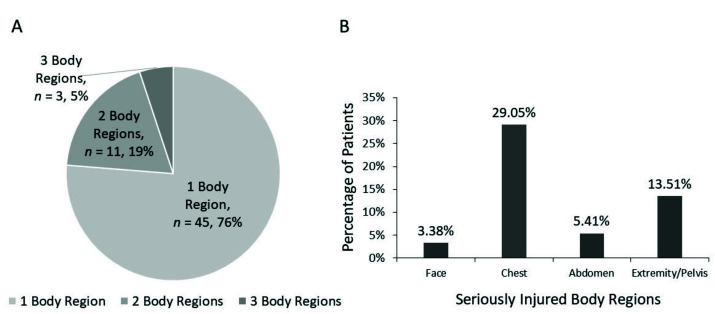
(**A**) The number of body regions with serious extracranial injuries (**B**) The distribution of seriously injured body regions.

There was a total of 148 children with sTBI recruited (Figure 1). Most of them were boys (74.32%). Their median age was 17 years old. Traffic accidents were the most common mechanism of injury (79.73%). Fifty-eight sTBI children (39%) were with SEI (Table 1). Seventy-six patients had only one additional body region injured. Among them, the chest was the most commonly associated with SEI, followed by the extremity/pelvis (Figure 2).

Compared to children without SEI, children with SEI were older (*p* < 0.001), and presented to ER with more hypotension (*p* = 0.003) and hypothermia (*p* = 0.005); initial lab showed more prolonged prothrombin time (*p* = 0.004) and had higher in-hospital mortality rate (*p* = 0.049) (Table 1). There were no significant differences in gender, mechanism of injury, initially presented motor component of GCS, pupil size, pupil reaction, Rotterdam CT score, and blood glucose level between the two groups of children.

Twenty-nine (19.6%) recruited sTBI children died in hospital. (Table 2) Compared to children who survived, sTBI children with in-hospital mortality had significant differences in the mechanism of injury (*p* = 0.026), more combined with SEI (*p* = 0.046); presented to ED having a lower motor component of GCS (*p* < 0.01), higher pupil size points (*p* = 0.003), lower pupil reaction points (*p* < 0.01); higher incident rate of hypotension (*p* < 0.01), hypothermia (*p* < 0.01), initial lab data had more common prolonged prothrombin time (*p* < 0.01) and hyperglycemia (*p* < 0.01) and initial brain CT findings had higher Rotterdam CT score (*p* < 0.01). There were no significant differences in gender, age, and whether they received neurosurgery or not between those two groups of patients.

In multivariate analyses of the potential predictors of in-hospital mortality among sTBI, mechanism of injury, coexisting SEI, motor component of GCS score, pupil size, pupil reaction, hypotension, hypothermia, prolonged initial prothrombin time, initial hyperglycemia, Rotterdam CT score were evaluated (Table 3). There were the most missing values in hyperglycemia (11.5%), followed by pupil reaction (7.4%), pupil size (6.8%), Rotterdam CT score (6.8%), and prothrombin time (4.1%). We identified motor components of GCS (adjusted OR = 1.78, 95% CI: 1.2–2.8, *p* = 0.01), fixed pupil reaction (adjusted OR = 5.68, 95% CI: 1.2–28.0, *p* = 0.033), prolonged prothrombin time (adjusted OR = 6.57, 95% CI: 1.0–43.0, *p* = 0.049) and Rotterdam CT score (adjusted OR = 2.12, 95% CI: 1.1–4.0, *p* = 0.023) were independent predictors of in-hospital mortality. SEI was not an independent predictor of mortality.

## 4. Discussion

In our study, 39% of recruited sTBI children had coexisting SEI. Severe TBI children with SEI were older, more presented to ED with hypothermia and hypotension, and initial laboratory findings showed more prolonged prothrombin time and higher in-hospital mortality when compared with those without SEI. Multivariate analysis showed SEI was not an independent predictor of mortality in these severe TBI children. 

Coexisting SEI is common in sTBI patients who suffer high-impact injury. Most sTBI children with SEI had one body region other than the brain involved. Chest and pelvis/ extremities injuries were the most common in SEI regions [12,14]. Severe TBI children with SEI were older when compared to those without SEI. Older adolescents may more commonly suffer higher impact injuries compared to young children. High impact trauma energy may lead to not only sTBI but additional extracranial injuries. Besides, people between 15–24 years old were more vulnerable to road traffic accidents, which were high-impact/speed injuries, and more related to non-isolated TBI [26]. Although road traffic collisions were the most common injury mechanism of sTBI with SEI, our study suggested there were no significant differences in mechanisms of injury between those with SEI or without SEI. High-energy transfer injuries, including fall from height, traffic accidents, violence, and suicide, may lead to SEI in sTBI children. 

Our study showed that sTBI children with SEI initially presented more with hypotension and hypothermia than those without SEI. Initial hypotension and hypothermia were associated with increased mortality in all sTBI children. A previous study had suggested that even isolated sTBI children may also commonly present with initial hypotension and hypothermia and was associated with poor outcomes [22,23,27]. SEI may cause blood loss, hemorrhagic shock, and subsequent peripheral vasoconstriction and tissue hypoperfusion, resulting in systemic changes, including hypotension and hypothermia. These physiologic insults in pediatric sTBI, who have impaired autoregulation of brain blood flow, further lead to reduced cerebral blood flow, secondary brain injury, and increased mortality [28,29,30].

sTBI children with SEI had a significantly higher incidence of initial prolonged prothrombin time in our study. Initial prolonged prothrombin time was associated with higher mortality in sTBI children and was an independent predictor of mortality in multivariate analysis. Such findings are compatible with previous studies [27,31,32]. Following severe traumatic injury, hypothermia, acidosis, hemodilution, and consumption of coagulation factors, secondary to local activation of the coagulation system, result in coagulopathy [31]. Coagulopathy presenting as a prolonged international normalized ratio (INR) likely serves as a marker of systemic dysregulation [33]. Although no evidence showed that correction of trauma-induced coagulopathy could improve outcome [32], early monitoring of the sTBI children’s coagulation profile can be used to predict the outcome [33].

We identified that SEI was associated with increased mortality in sTBI children compared to those without SEI. Studies by Tanya Chark Stewart et al. and Mohamed Afiq Muizz Mohamed Rasidi et al. suggested SEI was not associated with increased mortality in sTBI children [12,14]. The different results may relate to the more severe brain injury intensity in the SEI groups compared to those without SEI in their studies. A collaborative analysis of a large number of TBI patients suggests SEI is a prognostic factor for increased mortality in TBI, but the strength of the effect is smaller in patients with a more severe brain injury which may explain the differences [15]. Keita Shibahashi et al. [13] analyzed the effects of SEI on in-hospital mortality and used 15 variables in multivariate logistic regression analysis, including: adolescent; year of admission; gender; GCS on arrival; hypotension on arrival; cause of trauma; head injury type including subdural hemorrhage; epidural hemorrhage; contusion; intracerebral hemorrhage; diffuse axonal injury; vault fracture; base fracture; underwent craniotomy; and SEI. They identified that SEI not only led to significantly higher mortality but was an independent predictor of mortality in sTBI children. We also identified that sTBI children with SEI had significantly higher mortality than those without SEI. However, after controlling for the confounding effects of all other variables, SEI was not an independent predictor of mortality in our study. Such difference may be related to the use of early recognized physiologic responses to severe injury, including hypothermia, hyperglycemia, prolonged prothrombin time, and hypotension in the multivariate logistic regression in our study. We identified motor components of GCS, fixed pupil reaction, prolonged prothrombin time, and higher Rotterdam CT score as independent predictors of mortality. It has been suggested that the effect of SEI on mortality in TBI may not only be caused by the direct influence of SEI but also an inflammatory response to severe injuries or the worsening effect on the brain injury itself caused by hypovolemia or ischemia [15]. Our findings suggest that the severity of brain injury in pediatric sTBI and its presentations are still the most important predictors of in-hospital mortality. SEI itself did not independently predict in-hospital mortality, but the severe injury-associated systemic response, which presented as initial prolonged prothrombin time, did predict in-hospital mortality independently. Increased mortality in sTBI children with SEI may be related to the synergistic effect of sTBI and SEI but not the direct effect of SEI [13].

### Limitation

This study has some limitations. First, this was a single-center, retrospective cohort study. Despite that, we believe our data were relevant to other medical centers as we used standard criteria to include sTBI patients (initial GCS ≤ 8) and SEI (AIS ≥ 3). However, the sample population was limited and may not be representative of the whole population. Further study with a larger sample size was needed. Second, due to the nature of the study design, the selection bias and missing data could not be completely prevented. We used multiple imputations for the missing data since valid multiple imputations can reduce bias even when the proportion of missing data is large [34]. Third, not all potential predictors for in-hospital mortality were examined in this study, as we only assessed routinely documented and well-recorded clinical characteristics. The magnitude of the accidents and vehicles involved, use of protective devices or not, alcohol and drug use when injured, and details of resuscitation were lacking. Finally, the long-term outcome was not assessed in this study due to the limit of the retrospective study. Further long-term, prospective cohort studies involving more identifiable risk factors are warranted to clarify the influence of SEI on sTBI children.

## 5. Conclusions

Severe TBI children combined with SEI were associated with higher in-hospital mortality than those without SEI. SEI was not an independent predictor of mortality in sTBI children. The severity of brain injury and its presentations, including motor GCS score, pupil reaction, and Rotterdam CT scores, were still the most important predictors of in-hospital mortality. Although SEI did not predict in-hospital mortality independently, severe injury-induced systemic response, which presented as initial prolonged prothrombin time, was another independent predictor of in-hospital mortality in these sTBI children.

## Figures and Tables

**Table 1:** Demographic and clinical characteristics of total, with and without serious extracranial injury.

Clinical Characteristics	Total (*n* = 148)	With SEI (*n* = 58)	Without SEI (*n* = 90)	*p* Value
Patient Characteristics:				
Gender, *n* (%)				
Boys	110 (74.32)	44 (75.86)	66 (73.33)	0.731
Girls	38 (25.68)	14 (24.14)	24 (26.67)	
Age (years)				
Median (25, 75%)	17 (8.5,18)	17 (16,18)	15 (5, 18)	<0.001
Mechanism of injury, *n* (%)				
Fall	17 (11.49)	3 (5.17)	14 (15.56)	0.080
Against	2 (1.35)	0 (0)	2 (2.22)	
Traffic accident	118 (79.73)	50 (86.21)	68 (75.56)	
Insult	9 (6.08)	3 (5.17)	6 (6.67)	
Suicide	2 (1.35)	2 (3.45)	0 (0)	
Motor component of GCS				
1	31 (20.95)	16 (27.59)	15 (16.67)	0.321
2	6 (4.05)	3 (5.17)	3 (3.33)	
3	5 (3.38)	3 (5.17)	2 (2.22)	
4	42 (28.38)	13 (22.41)	29 (32.22)	
5	64 (43.24)	23 (39.66)	41 (45.56)	
Pupil size				
0	79 (56.83)	28 (52.83)	51 (59.30)	0.755
1	19 (13.67)	8 (15.09)	11 (12.79)	
2	41 (29.50)	17 (32.08)	24 (27.91)	
Pupil reaction				
0	94 (68.61)	32 (60.38)	62 (73.81)	0.255
1	10 (7.30)	5 (9.43)	5 (5.95)	
2	33 (24.09)	16 (30.19)	17 (20.24)	
Rotterdam CT score				
1	4 (2.88)	0 (0)	4 (4.65)	0.132
2	26 (18.71)	7 (13.21)	19 (22.09)	
3	39 (28.06)	15 (28.30)	24 (27.91)	
4	31 (22.30)	17 (32.08)	14 (16.28)	
5	26 (18.71)	8 (15.09)	18 (20.93)	
6	13 (9.35)	6 (11.32)	7 (8.14)	
Hypotension				
Present	11 (7.43)	9 (15.52)	2 (2.22)	0.003
No present	137 (92.57)	49 (84.48)	88 (97.78)	
Hypothermia				
Present	19 (12.84)	13 (22.41)	6 (6.67)	0.005
No present	129 (87.16)	45 (77.59)	84 (93.33)	
Prothrombin time				
>1.2	24 (16.90)	16 (28.07)	8 (9.41)	0.004
≤1.2	118 (83.10)	41 (71.93)	77 (90.59)	
Blood glucose				
>200	45 (34.35)	20 (36.36)	25 (32.89)	0.680
≤200	86 (65.65)	35 (63.64)	51 (67.11)	
Mortality, *n* (%)				
Yes	29 (19.59)	16 (27.59)	13 (14.44)	0.049
No	119 (80.41)	42 (72.41)	77 (85.56)	

SEI: serious extracranial injury; GCS: Glasgow Coma Scale; CT: Computed tomography.

**Table 2:** Univariate analyses of association of mortality in children with severe traumatic brain injury.

	Alive (*n* = 119) *n* (%)	Die (*n* = 29) *n* (%)	Test Statistic	*p* Value
Patient Characteristics:				
Gender				
Boys	89 (80.91)	21 (19.09)	0.069	0.793
Girls	30 (78.95)	8 (21.05)		
Age (years)				
Median (25, 75%)	17.5 (16.18)	17.5 (16.18)		0.908
Mechanism of injury				
Fall	14 (82.35)	3 (17.65)	11.024	0.026
Against	0 (0)	2 (100)		
Traffic accident	98 (83.05)	20 (16.95)		
Insult	6 (66.67)	3 (33.33)		
Suicide	1 (50)	1 (50)		
Clinical Presentations:				
Serious extracranial injury				
SEI	42 (72.41)	16 (27.59)	3.866	0.049
No SEI	77 (85.56)	13 (14.44)		
Motor component of GCS				
5	59 (49.58)	5 (17.24)	43.631	<0.001
4	39 (32.77)	3 (10.34)		
3	5 (4.20)	0 (0)		
2	3 (2.52)	3 (10.34)		
1	13 (10.92)	18 (62.07)		
Pupil size				
0	69 (84.34)	10 (12.66)	11.708	0.003
1	16 (84.21)	3 (15.79)		
2	25 (60.98)	16 (39.02)		
Pupil reaction				
0	86 (91.49)	8 (8.51)	33.354	<0.001
1	9 (90)	1 (10)		
2	15(45.45)	18 (54.55)		
Hypotension				
Present	4 (36.36)	7 (63.64)	14.630	<0.001
Not present	115(83.94)	22 (16.06)		
Hypothermia				
Present	9 (47.37)	10 (52.63)	15.101	<0.001
Not present	110 (85.27)	19 (14.73)		
Prothrombin time				
>1.2	10 (41.67)	14 (58.33)	27.205	<0.001
≤1.2	104 (88.14)	14 (11.86)		
Blood glucose				
>200	28 (62.22)	17 (37.78)	12.346	<0.001
≤200	76 (88.37)	10 (11.63)		
Rotterdam CT score				
1	4 (100)	0 (0)	32.379	<0.001
2	22 (84.62)	4 (15.38)		
3	38 (97.44)	1 (2.56)		
4	27 (87.10)	4 (12.90)		
5	19(73.08)	7 (26.92)		
6	4 (30.77)	9 (69.23)		

GCS: Glasgow Coma Scale; CT: Computed tomography.

**Table 3:** Multivariate predictive models for mortality in children with severe traumatic brain injury.

	Adjusted OR	95% CI	z Score	*p* Value
Mechanism				
Fall	1			
Traffic accident	0.20	0.02–1.62	−1.51	0.130
Insult	0.73	0.06–9.47	−0.24	0.809
Suicide	2.72	0.00–1863.25	0.30	0.764
Serious extracranial injury	1.93	0.44–8.44	0.87	0.383
Motor component of GCS	1.78	1.15–2.76	2.58	0.010
Pupil size				
Bilaterally not dilated	1			
Anisocoric	0.40	0.04–3.59	−0.82	0.414
Bilaterally dilated	1.33	0.24–7.48	0.32	0.745
Pupil reaction				
Both constricted	1			
Inconsistent	0.43	0.02–9.57	−0.54	0.590
Fixed	5.68	1.15–28.04	2.13	0.033
Hypotension	1.45	0.14–15.35	0.31	0.759
Hypothermia	0.31	0.04–2.43	−1.11	0.266
Prothrombin time	6.57	1.00–42.99	1.97	0.049
Hyperglycemia	1.92	0.43–8.56	0.86	0.391
Rotterdam CT score	2.12	1.11–4.04	2.28	0.023

GCS = Glasgow Coma Scale Score; CT = Computed tomography; OR = Odds ratio; CI = Confidence interval.

## Data Availability

The data presented in this study are available on request from the corresponding author. The data are not publicly available due to the restriction of local law and government policy.
